# The Association between Continuity of Care and All-Cause Mortality in Patients with Newly Diagnosed Obstructive Pulmonary Disease: A Population-Based Retrospective Cohort Study, 2005-2012

**DOI:** 10.1371/journal.pone.0141465

**Published:** 2015-11-03

**Authors:** Kyoung Hee Cho, Young Sam Kim, Chung Mo Nam, Tae Hyun Kim, Sun Jung Kim, Kyu-Tae Han, Eun-Cheol Park

**Affiliations:** 1 Department of Public Health, Graduate School, Yonsei University, Seoul, Korea; 2 Institute of Health Services Research, College of Medicine, Yonsei University, Seoul, Korea; 3 Department of Internal Medicine, College of Medicine, Yonsei University, Seoul, Korea; 4 Department of Biostatistics, College of Medicine, Yonsei University, Seoul, Korea; 5 Graduate School of Public Health, Yonsei University, Seoul, Korea; 6 Department of Preventive Medicine, College of Medicine, Yonsei University, Seoul, Korea; Texas Tech University Health Science Centers, UNITED STATES

## Abstract

**Background:**

The disease burden is increasing for chronic obstructive pulmonary disease (COPD) due to increasing of the growth rate of prevalence and mortality. But the empirical researches are a little for COPD that studied the association between continuity of care and death and about predictors effect on mortality.

**Objective:**

To investigate the association between continuity of care (COC) and chronic obstructive pulmonary disease (COPD) mortality and to identify other mortality-related factors in COPD patients.

**Methods:**

We conducted a longitudinal, population-based retrospective cohort study in adult patients with COPD from 2002 to 2012 using a nationwide health insurance claims database. The study sample included individuals aged 40 years and over who developed COPD in 2005 and survived until 2006. We performed a Cox proportional hazard regression analysis with COC analyzed as a time-dependent covariate.

**Results:**

Of the 3,090 participants, 60.8% died before the end of study (N = 1,879). The median years of survival for individuals with high COC (COC index≥0.75) was 3.92, and that for patients with low COC (COC index<0.75) was 2.58 in a Kaplan Meier analysis. In a multivariate, time-dependent analysis, low COC was associated with a 22% increased risk of all-cause mortality (HR, 1.22; 95% CI, 1.09–1.36). Not receiving oxygen therapy at home was associated with a 23% increased risk of all-cause mortality (HR, 1.23; 95% CI, 1.01–1.49). Moreover, the risk of all-cause mortality for individuals who admitted one time increased 38% (HR, 1.38; 95% CI, 1.21–1.59), two times was 63% (HR, 1.63; 95% CI, 1.34–1.99) and 3+ times was 96% (HR, 1.96; 95% CI, 1.63–2.36) relative to the reference group (no admission).

**Conclusions:**

High COC was associated with a decreased risk of all-cause mortality. In addition, home oxygen therapy and number of hospital admissions may predict mortality in patients with COPD.

## Introduction

As many societies age, the prevalence of chronic diseases is increasing. The burden of disease is shifting from communicable diseases to non-communicable diseases in developed countries. Policymakers worldwide are beginning to recognize the magnitude of the problem. To efficiently manage chronic diseases with limited resources, policymakers are becoming interested in the benefits of continuity of care (COC), which can reduce the risk of complications [1], improve preventive care [2, 3], increase patients satisfaction [4] and compliance [5, 6], and decrease emergency and inpatient medical services and care costs [7–12]. In primary care, especially, COC is being introduced to improve quality and to cope with the increased workload associated with chronic diseases [13].

Continuity can be defined from various perspectives: informational continuity; management continuity; and relational continuity, or an ongoing therapeutic relationship between a patient and one or more providers [14]. Relational continuity, in particular, can reinforce care in patients with chronic conditions, because a continuous provider is more likely to know when tests are needed and treatment changes are indicated [15]. Relational continuity refers to a special type of longitudinal continuity; the latter implies a pattern of visits but does not directly address the nature of the relationship between patient and provider [16].

The Korean health care delivery system is classified into three steps based on fee-for-service as the reimbursement mechanism: clinics function as primary care institutions, hospitals function as secondary care institutions, and general hospitals and tertiary general hospitals function as third tier care institutions. All of these can provide outpatient services. Korea’s system is quite different from the managed care delivery system of the US where a patient’s selection of health care provider is regulated and restricted. Above all, because most clinical practitioners in Korea are specialists, they do not perform the function of a primary care physician as seen in the US [17]. In Korea, primary care physicians typically work in solo private practices and are reimbursed on a fee-for-service basis. In terms of outpatient services, clinics compete against other clinics, hospitals, and some general hospitals. Given the context, universal coverage provided by National Health Insurance has improved the accessibility of medical care but this system enables patients to choose freely and lead to the high number of fragmented visits.

Chronic obstructive pulmonary disease (COPD) is one common chronic disease, and its prevalence continues to rise. Worldwide, disability-adjusted life years (DALYs) associated with COPD increased 45.9% from 1990 to 2010 [18]. In Korea, the incidence of chronic lower respiratory diseases increased 11.2% from 2011 to 2012 [19], and the prevalence of COPD increased from 2.5 per 100,000 people in 2007 to 7.2 people per 100,000 in 2012. Many previous studies have found associations between COC and health outcomes such as diabetes mellitus, hypertension, pediatric asthma, and some psychiatric disorders. There are fewer studies on COPD on diseases such as diabetes or hypertension, however. Moreover, studies involving long-term follow-up of COPD patients and survival are rare. Previous studies of COPD have shown that age, forced expiratory volume in one second (FEV_1_)_,_ body mass index, oxygen therapy, smoking history, number of previous hospital admissions, and number of emergency department visits are strongly associated with mortality [20–23].

The first aim of this study was to measure COC over 7 years in patients with COPD and to investigate the association between COC and mortality. The second aim was to examine other potential predictors of mortality in COPD patients, such as oxygen therapy and number of previous hospitalizations.

## Methods

### Data and study design

This study used the Korean National Health Insurance Service (KNHIS) claims database for 2002–2012. We conducted a retrospective cohort study of new adult COPD patients to investigate the association between continuity of ambulatory care and mortality over the 7-year follow up period. Participants were 40 years of age or older with newly diagnosed COPD, codes J43 and J44 (International Classification of Disease, 10^th^ edition [ICD-10]). That the diagnosis was new was confirmed by the lack of COPD claims in 2002–2004 and a first COPD claim in 2005. We measured continuity of ambulatory care for 7 years, and observed mortality from 2006 to 2012. Ethical approval for this study was granted by the Institutional Review Board of the Graduate School of Public Health, Yonsei University. The requirement for informed consent was waived because the study was based on routinely collected administrative and claims data. Patient information and records was de-identified prior to analysis.

### Study population

The total number of individuals with COPD 40 years or older was 1,538,711 during 2002–2012. Of these, 138,680 patients received their diagnosis in 2005. We excluded 135,590 patients with less than four outpatient visits per year in 2006–2012. This criterion was intended to facilitate calculation of the COC index in a structurally reasonable and meaningful manner. Another rationale for this criterion was to improve the accuracy of COPD diagnosis. We obtained COPD diagnoses from data, which are based on information from the KNHIS claims database. The accuracy of diagnosis in KNHIS claims data is roughly 70% [24]. In our study, we defined ‘outpatient visit’ that COPD patient only visited due to COPD as ICD-10 code, J43 or J44. We excluded visits which patient utilized outpatient services due to diabetes, hypertension, or the other diseases. When calculating COC index, we limited outpatient visits due to COPD. Thus, we needed a process to aid in the selection of participants that actually had COPD. Frequent COPD-related visits ensured that we included only patients with a valid diagnosis. Using these criteria, the final study sample included 3,090 participants.

### Variables

The dependent variable in this study was mortality. We defined mortality to include all causes of death, on the basis of information recorded in the KNHIS database.

We defined continuity as longitudinal continuity in this study. Thus, we calculated the COC index in subjects with four or more ambulatory care visits per year [25]. This is because in a country where over 90% of clinical practitioners are specialists, during 2006–2012, we measured the COC for each patient using the COC index. The COC index reflects the distribution of visits to different healthcare institutions. It is influenced by both the total number of providers and the total number of visits. The index ranges from 0 to 1; a higher value corresponds to better COC. The formula for the COC index is as follows:

$$\text{coc}\;=\frac{\sum \;{n_{j}}^{2}-N}{\text{N}(\text{N}-1)}$$ (1)

where N is the total number of ambulatory care visits, *n*_*j*_ is the number of visits to the *j*th provider. In this study, ‘potentially available providers’ refers not to medical physicians but to healthcare institutions. A COC score of 1 indicates that all visits were to the same provider. We defined high COC as a score >0.75, consistent with a previous study [26].

Covariates considered included age, sex, health insurance status (national health insurance, medical aid), Charlsons’ comorbidity index (0, 1, 2^+^), home oxygen therapy (yes, no), use of intensive care unit (ICU) medical service (yes, no), number of hospital admissions (0, 1, 2, 3^+^), and respiratory impairment grade (1, 2, 3, none); all of these variables were measured at the 2006 baseline. In the case of health insurance, people can qualify for medical aid if their household income is less than $600 per month based on single household. Respiratory impairment grade was defined using %FEV_1_, Pa,O_2_ and dyspnea. Grade 1 was defined as patients with an %FEV_1_ lower than 25% or Pa,O_2_ less than 55 mmHg while having difficulty breathing at rest; Grade 2 was defined as patients with %FEV_1_ lower than 30% or Pa,O_2_ less than 60mmHg while having difficulty breathing at moving in the home; Grade 3 was defined as patients with %FEV_1_ lower than 40% or Pa,O_2_ less than 65 mmHg while having difficulty at walking on flat. Respiratory impairment grade was defined using FEV_1_ and dyspnea. Only the comorbidity component of Charlsons’ comorbidity index was calculated, and all diagnostic information was collected from inpatient and outpatient billing data for 2006.

### Statistical analysis

In this analysis, participants were censored based on survival to the end of the study. We divided participants into two groups based on censored status, and compared the distributions of each variable of interest between the groups using chi-square and t-tests. Unadjusted median years of survival were estimated using the Kaplan-Meier method, and adjusted median years of survival were estimated using the Cox proportional hazard model with the Breslow method. This type of model was also used to identify factors associated with mortality. To avoid time-dependent bias [27], the COC index was treated as a time-dependent variable over the 7-year observed mortality period. Thus, we constructed a Cox proportional hazard model with time-dependent covariates to model the effect of COC on mortality. In this model, the previous year’s COC index reflected the next year’s survival. The log of cumulative hazard rates was proportional to the follow-up time, and no violations of the proportional hazard assumption were detected. All statistical analyses were performed using SAS statistical software (version 9.3).

## Results

In this study, 60.8% of participants died prior to the end of the study. Table 1 presents the characteristics of the study sample at baseline (2006), stratified by censored status. The mean age of the sample was 69.0 years. The two groups were significantly different with respect to all characteristics measured, except for sex, home oxygen therapy, and ICU use.

**Table 1:** Baseline characteristics of the study population according to death.

	All patients	Without death		With death		P-value
Characteristics	(N = 3,090)	(N = 1,211)		(N = 1,879)		
Age (years), mean (SD)	69.0 (10.1)	64.4	(9.3)	71.9	(9.5)	< .0001
Sex, n (%)						
Male	2,348	930	(76.8)	1,418	(75.5)	0.398
Female	742	281	(23.2)	461	(24.5)	
Health insurance type, n (%)						
National health insurance	1,857	768	(41.4)	1,089	(58.6)	0.003
Medical aid	1,233	443	(35.9)	790	(64.1)	
Home oxygen therapy, n (%)						
Yes	182	69	(37.3)	116	(62.7)	0.586
No	2,908	1142	(39.3)	1,763	(60.7)	
Charlson comorbidity index, n (%)^a^						
0	2,180	459	(39.6)	699	(60.4)	0.107
1	708	687	(39.7)	1,043	(60.3)	
≥2	202	65	(32.2)	137	(67.8)	
ICU use, n (%)						
Yes	46	12	(26.1)	34	(73.9)	0.067
No	3,044	1,199	(39.4)	1,845	(60.6)	
Number of hospital admission, n (%)						
0	2,416	1,037	(42.9)	1,379	(57.1)	< .0001
1	347	96	(27.7)	251	(72.3)	
2	157	43	(27.4)	114	(72.6)	
≥3	170	35	(20.6)	135	(79.4)	
Respiratory impairment grade, n (%)						
Grade 1 (%FEV_1_≤25 or Pa,O2 <55 mmHg)	168	69	(41.1)	99	(58.9)	0.016
Grade 2 (%FEV_1_≤35 or Pa,O2 <60 mmHg)	172	74	(43.0)	98	(57.0)	
Grade 3 (%FEV_1_≤40 or Pa,O2 <65 mmHg)	185	91	(49.2)	94	(50.8)	
None (%FEV_1_ and Pa,O2 = Normal)	2,565	977	(38.1)	1,588	(61.9)	

ICU, Intensive Care Unit; %FEV_1_, FEV_1_ predicted

^a^Calculated comorbidity scores; extracted age scores.

**Fig 1:**
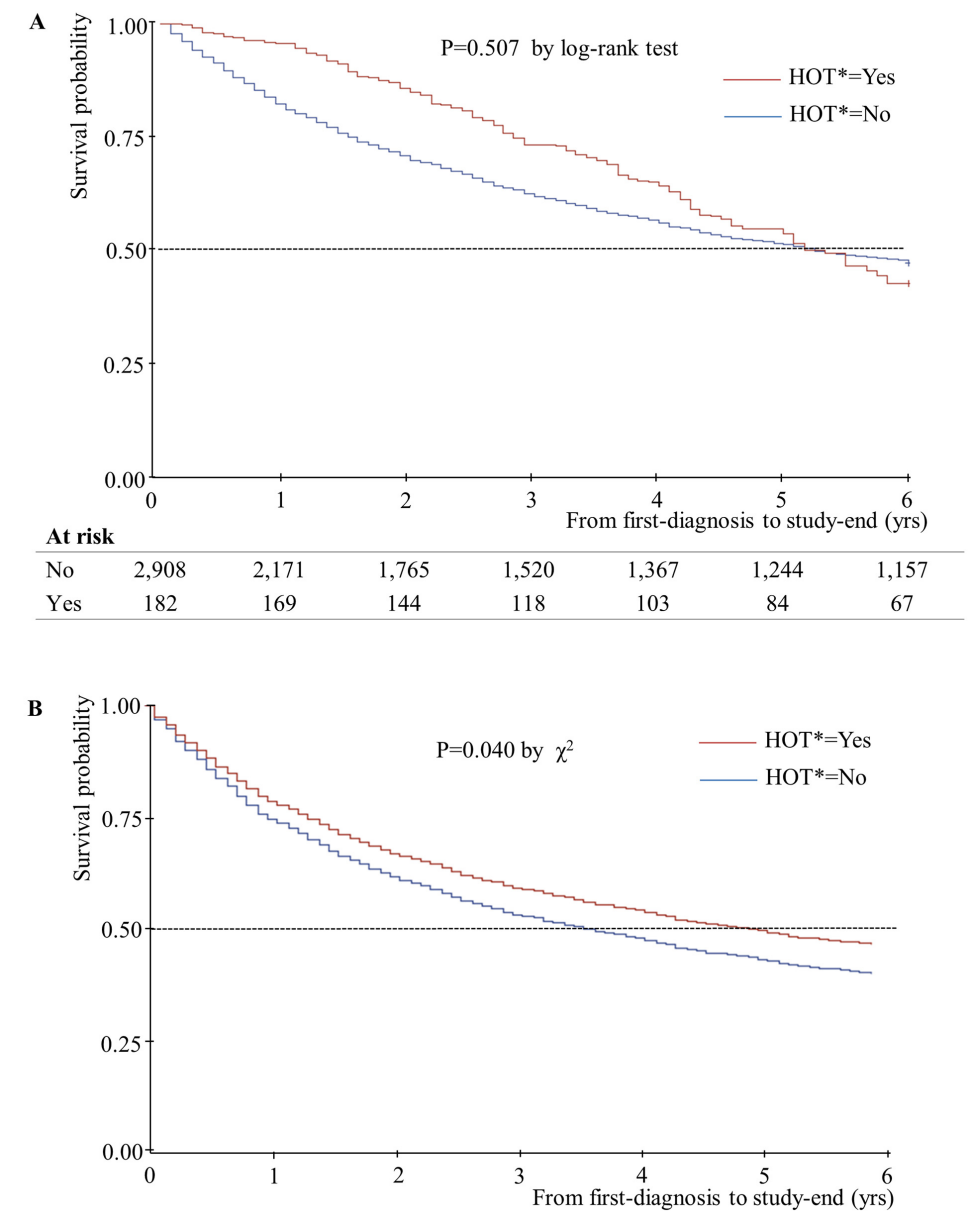
Median years of survival, stratified according to home oxygen therapy status (n = 3,090). (A) Kaplan-Meier survival plot for patients with newly diagnosed COPD over 6 years of follow-up: HOT* = No-Median 5.08yrs; HOT* = Yes- Median 5.25 yrs (B) Adjusted Cox’s proportional hazards regression plot: HOT* = No- Median 3.58yrs; HOT* = Yes- Median 4.92 yrs. *, Home oxygen therapy.

**Fig 2:**
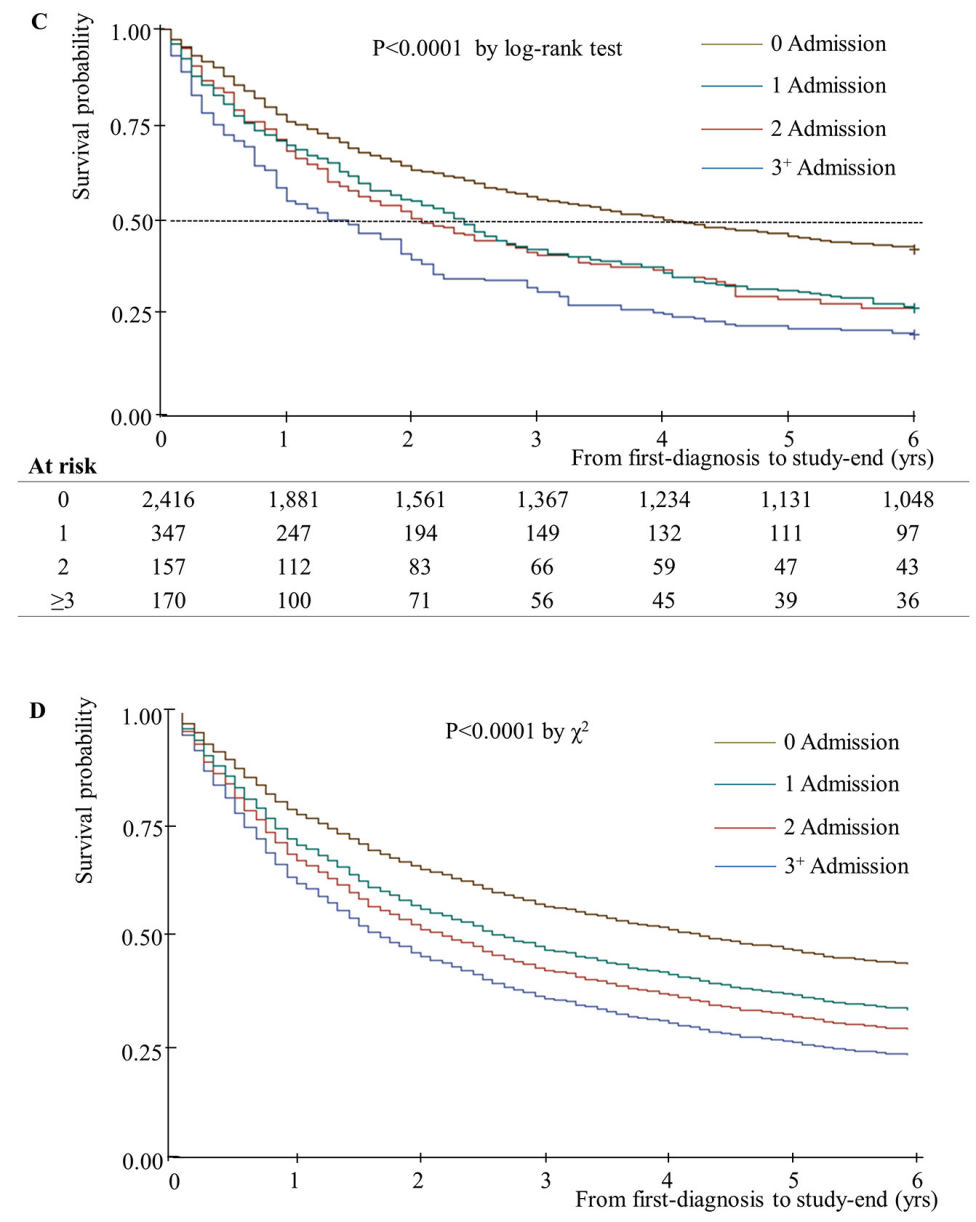
Median years of survival, stratified according to number of hospital admissions (n = 3,090). (C) Kaplan-Meier survival plot for patients with newly diagnosed COPD over 6 years of follow-up: 3^+^ Admission-Median 1.50 yrs; 2 Admission-Median 2.08 yrs; 1 Admission-Median 2.42 yrs; 0 Admission- Median 4.17 yrs. (D) Adjusted Cox’s proportional hazards regression plot: 3^+^ Admission-Median 1.65 yrs; 2 Admission- Median 2.17 yrs; 1 Admission-Median 2.58 yrs; 0 Admission-Median 4.25 yrs.

**Fig 3:**
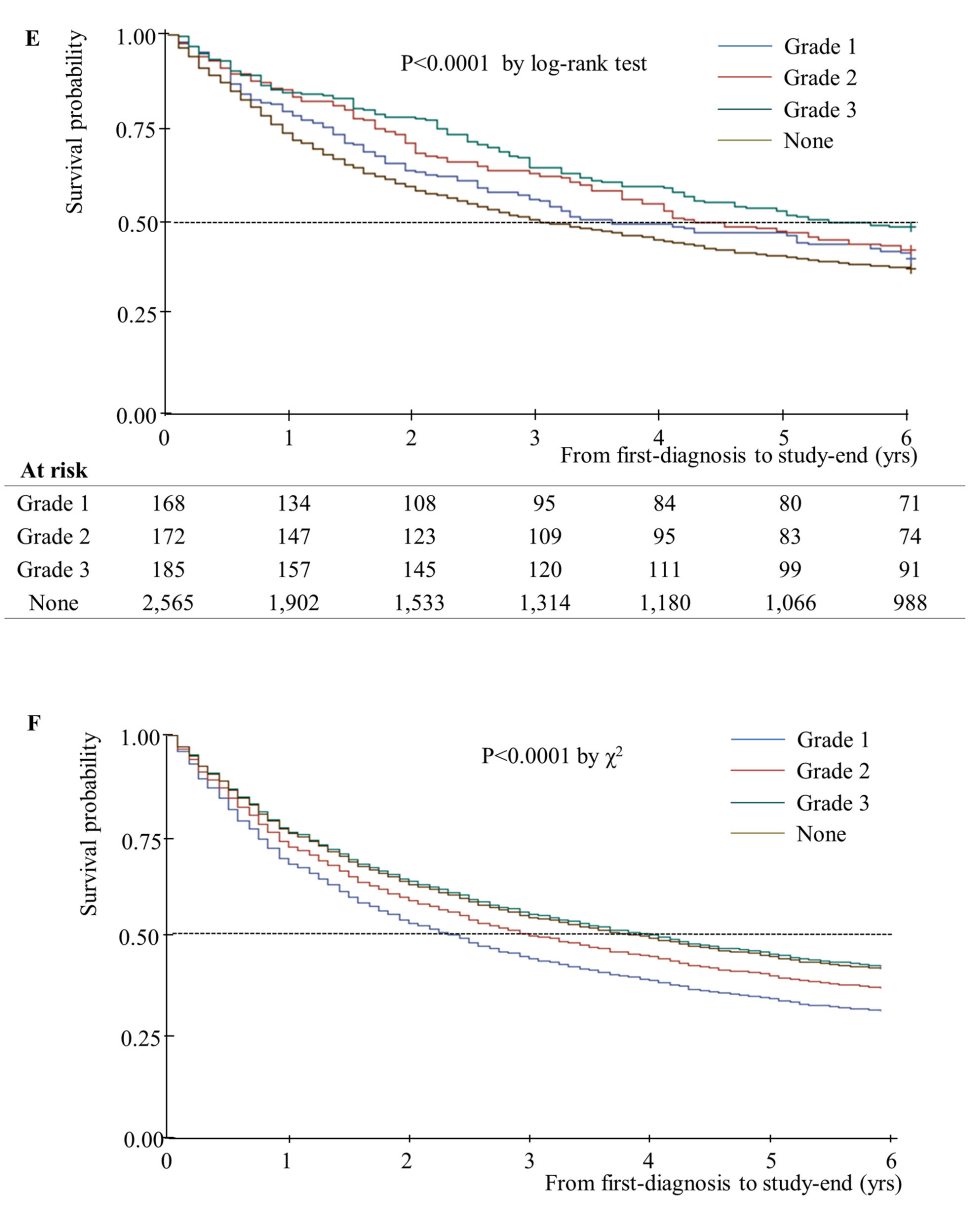
Median years of survival, stratified according to respiratory disability grade (n = 3,090). (E) Kaplan-Meier survival plot for patients with newly diagnosed COPD over 6 years of follow-up: Grade 1-Median 3.83 yrs; Grade 2-Median 4.50 yrs; Grade 3-Median 5.67 yrs; None-Median 3.17 yrs. (F) Adjusted Cox’s proportional hazards regression plot: Grade 1- Median 1.65 yrs; Grade 2-Median 2.17 yrs; Grade 3-Median 2.58 yrs; None-Median 4.25 yrs.

**Fig 4:**
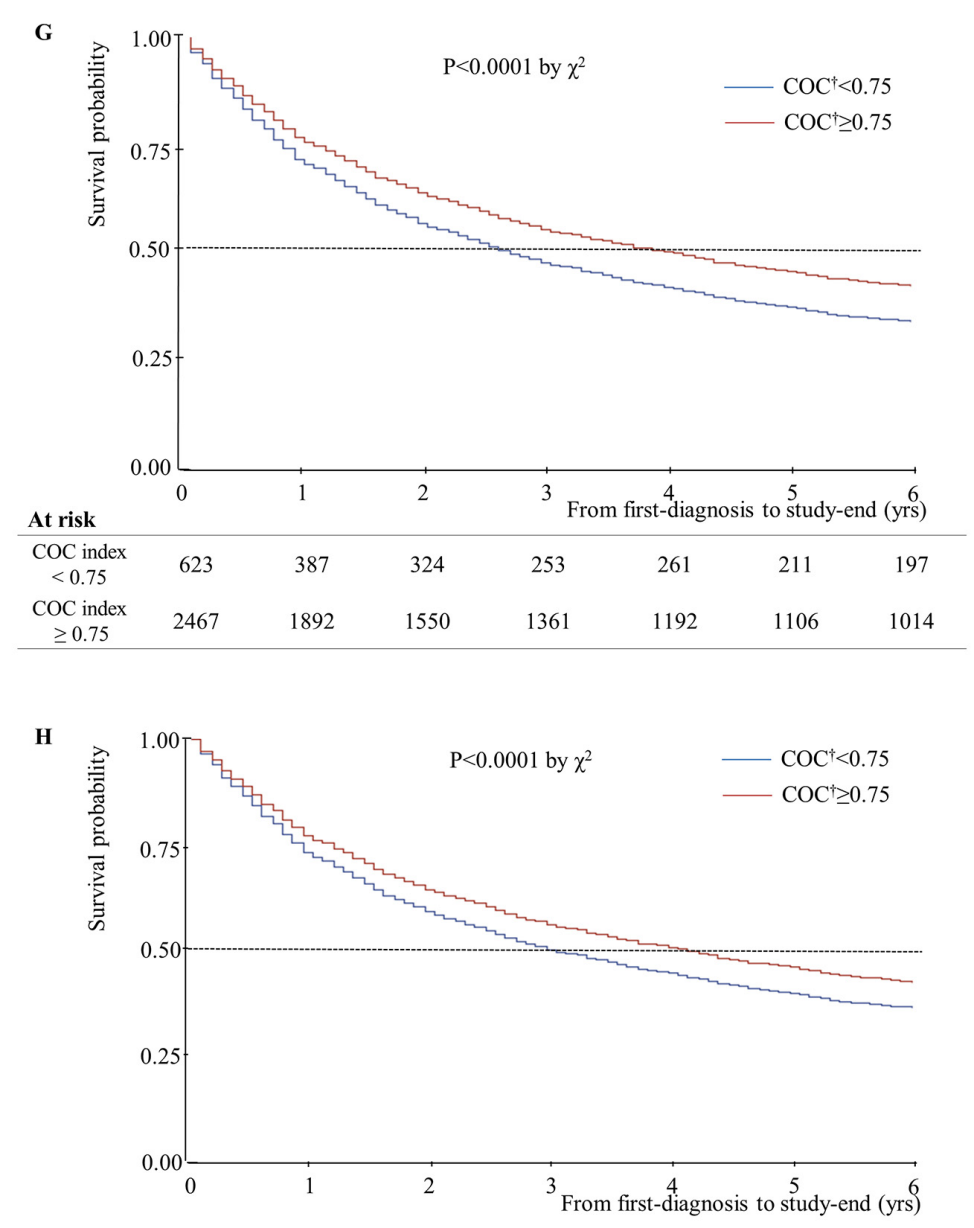
Median years of survival, stratified according to COC, which was treated as a time-dependent covariate (n = 3,090). (G) Plot for patients with newly diagnosed COPD over 6 years of follow-up using Breslow method; COC^†^ <0.75-Median 2.58 yrs; COC^†^ ≥0.75-Median 3.92 yrs. (H) Adjusted Cox’s proportional hazards regression plot: COC^†^ <0.75- Median 2.92 yrs; COC^†^ ≥0.75; Median 4.00 yrs. †, Continuity of care.

Unadjusted and adjusted median years of survival stratified by home oxygen therapy status are presented in Fig 1. The adjusted median years of survival for patients receiving home oxygen therapy was 4.92 years, and that for patients not receiving home oxygen therapy was 3.58 years. This difference was statistically significant (P = 0.04). In addition, unadjusted and adjusted median years of survival decreased as number of hospital admissions increased (Fig 2). Unadjusted median years of survival for 0/1/2/3^+^ hospital admissions were 4.17/2.42/2.08/1.50, respectively (P = 0.001), and adjusted median years of survival were 4.25/2.58/2.17/1.65 (P = 0.01). Unadjusted median years of survival were shortest for individuals with no respiratory impairment grade, at 3.17 years (Fig 3). However, after controlling for covariates, median years of survival were shortest for respiratory impairment grade 1 patients, at 2.33 years. Finally, unadjusted median years of survival for participants with high COC were greater than for participants with low COC (3.92 and 2.58 years, respectively), and adjusted median years of survival displayed the same relationship (4.00 and 2.92 years, respectively; Fig 4).

The mortality hazard ratio increased with age (HR, 1.06; 95% CI, 1.05–1.07) after controlling for covariates including age, sex, health insurance type, use of home oxygen therapy, Charlson comorbidity index, ICU use, Number of hospital admission, and respiratory impairment grade. In addition, the adjusted mortality hazard ratio was significantly higher for individuals who did not receive home oxygen therapy (HR, 1.21; 95% CI, 1.01–1.47), The adjusted hazard ratio also increased with the number of hospital admissions; the adjusted hazard ratios for individuals with 1, 2, and 3 or more admissions relative to the reference group (0 admissions) were 1.38 (95% CI, 1.20–1.58), 1.60 (95% CI, 1.31–1.96), and 1.94 (95% CI, 1.60–2.34), respectively. Moreover, relative to individuals with no respiratory disability, the adjusted hazard ratio was 1.42 (95% CI, 1.14–1.77) for those with grade 1 respiratory disability, 1.17 (95% CI, 0.94–1.45) for those with grade 2 disability, and 0.99 (95% CI, 0.80–1.22) for those with grade 3 disability. Finally, in a time-dependent Cox regression analysis adjusted for patient characteristics, COC was significantly associated with mortality. Low COC (COC index< 0.75) was associated with a 22% increased risk of all-cause death in any given year (adjusted HR, 1.22; 95% CI, 1.09–1.36; Table 2).

**Table 2:** Hazard ratio for death by Cox’s proportional model.

	HRs for 7-year mortality (95% CI)				
Characteristics	Unadjusted			Adjusted^a^	
Age (years)	1.06	(1.05–1.06)		1.06	(1.05–1.07)
Sex					
Male	091	(0.82–1.02)		1.06	(0.95–1.19)
Female	1.00			1.00	
Health insurance type					
National health insurance	1.00			1.00	
Medical aid	1.13	(1.03–1.25)		1.05	(0.95–1.15)
Home oxygen therapy					
Yes	1.00			1.00	
No	1.12	(0.92–1.35)		1.21	(1.01–1.47)
Charlson comorbidity index^b^					
0	1.00			1.00	
1	0.98	(0.89–1.08)		0.94	(0.85–1.04)
≥2	1.22	(1.01–1.47)		1.01	(0.83–1.22)
ICU use					
Yes	1.33	(0.95–1.87)		1.51	(1.07–2.14)
No	1.00			1.00	
Number of hospital admission					
0	1.00			1.00	
1	1.50	(1.31–1.72)		1.38	(1.20–1.58)
2	1.51	(1.24–1.83)		1.60	(1.31–1.96)
≥3	1.99	(1.66–2.38)		1.94	(1.60–2.34)
Respiratory impairment grade					
Grade 1 (%FEV_1_≤25 or Pa,O_2_ <55 mmHg)	0.90	(0.73–1.10)		1.42	(1.14–1.77)
Grade 2 (%FEV_1_≤35 or Pa,O_2_ <60 mmHg)	0.82	(0.66–1.01)		1.17	(0.94–1.45)
Grade 3 (%FEV_1_≤40 or Pa,O_2_ <65 mmHg)	0.71	(0.58–0.87)		0.99	(0.80–1.22)
None (%FEV_1_and Pa,O_2_ = Normal)	1.00			1.00	
COC index^c^					
High (COC index≥0.75)	1.00			1.00	
Low (COC index<0.75)	1.26	(1.13–1.41)		1.22	(1.09–1.36)

ICU, Intensive Care Unit; %FEV_1_, FEV_1_ predicted; COC, Continuity of Care.

^a^ adjusted for age, sex, age, sex, health insurance type, use of home oxygen therapy, Charlson comorbidity index, ICU use, number of hospital admissions, and respiratory impairment grade.

^b^ Calculated comorbidity scores; extracted age scores.

^c^ Time-dependent covariate; COC index ranged from 0 to 1; 1 means that one patient has visited only one physician.

## Discussion

We found that COPD patients with higher COC had a lower risk of mortality. Low COC was associated with 22% higher mortality. We also found that home oxygen therapy was associated with a 21% decreased risk of mortality and that risk of mortality increased with number of visits to the hospital admission department.

We could not find the previous research that investigated the association between COC and mortality in patients with COPD documented in this study. However, in perspective that high COC lead to good health outcome, our result is consistent with previous studies of COC and outcomes in COPD patients [12, 28]. Our study did not reveal the mechanism underlying these results, but there are some possible explanations in terms of two contrasted aspects. First, Gray et al. have suggested that COC could benefit patients by reinforcing accumulation of patient knowledge, improving interpersonal communication, and increasing the likelihood that patients adhere to their doctors’ advice [29]. Another study found that the benefits of continuity may be magnified in patients with chronic disease, because patients with chronic disease are more likely than healthy people to use outpatient services and may establish relationships with their physicians more quickly [30]. In another aspect, there was the possibility that patients with uncontrolled or severe COPD visited another institution, when their condition were doing poorly or were not stable. Because we used claims data, we could not distinguish that patients who visited several different institutions were whether their condition were worse or they did doctor shopping although their condition was stable.

Adjusted median survival time of patients receiving home oxygen therapy was 1.34 years longer than those without. An analysis of unadjusted median survival time indicated that the survival of patients with home oxygen therapy was better for the first 5 years, but afterwards survival of patients without home oxygen therapy was better. This result suggests that home oxygen therapy may be effective in prolonging survival. We controlled use of home oxygen therapy according to literature which home oxygen therapy was associated with better survival in COPD patients with severe hypoxemia such as Pa,O_2_ was 60 mmHg or less. Because home oxygen therapy is usually indication to severe COPD patients, home oxygen use might be a marker for much sicker patients with poorer expected survival. So we identified the association between home oxygen therapy use and survival stratified to respiratory impairments (S1 Table). Only in severe respiratory impairment group with grade 1 of respiratory disability, home oxygen therapy use was associated with decreasing the risk of all-cause mortality. In the other groups, there was not statistically significant difference.

This study has some limitations. The first is that we used all-cause deaths to measure mortality. We were not able to limit our analysis to only COPD-related deaths because cause of death was not included in the health insurance qualification database. As a result, mortality as measured in this study may not be directly related to continuity of ambulatory care received by COPD patients. The second limitation is the accuracy of COPD diagnosis. We used NPS data, which are based on information in the KNHI claims database. The accuracy of diagnosis in KNHI claims data is about 70%. To increase accuracy, a review of all prescriptions would be required. Unfortunately, we could not perform such a review. Thus, the accuracy of COPD diagnoses in this study may have been compromised. Also, because we only included the patients who visited more than 4 times due to COPD per each year to identify real COPD patients and calculate COC index, it might be included a little severe COPD patients and our results could not reflect the characteristics of patients with fewer than four ambulatory visits. The third limitation involves the definition of newly diagnosed patients. In this study, they were identified as patients who did not have COPD claims in 2002–2004 but had a COPD claim in 2005. Thus, patients diagnosed prior to 2002 that did not utilize COPD-related medical services in 2002–2004 may have been included in the sample. The fourth limitation is that we could not consider all factors that might affect the association between COC and mortality, such as body mass index, income level, education, residential area, and health behaviors, due to limitations of the claims database. Especially, health behaviors such as smoking and bronchospasm are highly related, but we could not identify. The fifth limitation is that our results do not reflect characteristics of patients with fewer than four ambulatory visits per year. We excluded such patients to increase the validity of our measurement of continuity. For example, in some cases where actual continuity was not good, a continuity value could be relatively high because a patient visited the same physician twice in a year. We excluded such individuals to optimize accurate estimation of COC. The final limitation is that we could not identify individual service providers on the basis of information from the claims database. Hence, in this study, outpatient healthcare provider was not a physician but a medical institution.

Despite these limitations, our study has several strengths. First, we analyzed COPD patients using nationwide claims data and conducted long-term follow up from 2005–2012. Although many patients were excluded to increase the validity of our COC measure, our study population was relatively large compared to previous studies evaluating the association between oxygen therapy and mortality [21, 22]. Furthermore, to avoid time-dependent bias, we used a longitudinal study design and treated COC as a time-dependent variable when investigating the association between continuity of ambulatory care and mortality. In addition, we included several proxy variables of health status in our models, such as ICU use, respiratory impairment grade, and number of visits to the hospital admission department. Inclusion of such variables may decrease bias due to confounding. Finally, one strength of this study was that we were able to increase the homogeneity of our study sample by identifying newly diagnosed patients.

In conclusion, our results support the hypothesis that improving COC can reduce mortality risk in patients with COPD. We should encourage policymakers to recognize the need for an effective healthcare delivery system that promotes COC by encouraging a team approach and enhancing accessibility.

## Conclusion

The risk of mortality for patients with COPD declines with increasing COC. In addition, home oxygen therapy and number of visits to the hospital admission department predict mortality in patients with COPD. Further research on the associations between COC and various healthcare outcomes, adjusting for potential confounders such as income, education, and health behaviors, is needed.

## Supporting Information

S1 Table Adjusted hazard ratio for 6-year mortality, according to respiratory disability grade. (DOCX)
