# Correction: The Association between Continuity of Care and All-Cause Mortality in Patients with Newly Diagnosed Obstructive Pulmonary Disease: A Population-Based Retrospective Cohort Study, 2005–2012

**DOI:** 10.1371/journal.pone.0148153

**Published:** 2016-01-25

**Authors:** Kyoung Hee Cho, Young Sam Kim, Chung Mo Nam, Tae Hyun Kim, Sun Jung Kim, Kyu-Tae Han, Eun-Cheol Park

The first and seventh authors, Kyoung Hee Cho and Eun-Cheol Park, should not have been listed as contributing equally to this work. The second and third authors, Young Sam Kim and Chung Mo Nam, should also not have been listed as contributing equally to this work.

## References

[1] Cho KH, Kim YS, Nam CM, Kim TH, Kim SJ, Han K-T, et al (2015) The Association between Continuity of Care and All-Cause Mortality in Patients with Newly Diagnosed Obstructive Pulmonary Disease: A Population-Based Retrospective Cohort Study, 2005–2012. PLoS ONE 10(11): e0141465 doi:10.1371/journal.pone.0141465 26529224 10.1371/journal.pone.0141465 PMC4631362

