# Supplementary material for: The Association between Continuity of Care and All-Cause Mortality in Patients with Newly Diagnosed Obstructive Pulmonary Disease: A Population-Based Retrospective Cohort Study, 2005-2012
Source: PLoS One. 2015 Nov 3;10(11):e0141465. doi: 10.1371/journal.pone.0141465 (PMC4631362; doi:10.1371/journal.pone.0141465)
Supplement: S1 Table — (DOCX) [file pone.0141465.s001.docx]

| S1 Table. Adjusted hazard ratio for 6-year mortality, according to respiratory disability grade | | | | | | | | | | | |
| --- | --- | --- | --- | --- | --- | --- | --- | --- | --- | --- | --- |
|  | **Adjusted HRs for 7-year mortality (95% CI)^*^** | | | | | | | | | | |
| Chracteristics | Grade 1 | |  | Grade 2 | |  | Grade 3 | |  | None | |
| Home oxygen therapy |  |  |  |  |  |  |  |  |  |  |  |
| Yes | 1.00 |  |  | 1.00 |  |  | 1.00 |  |  | 1.00 |  |
| No | 1.98 | (1.10-3.58) |  | 0.91 | (0.49-1.68) |  | 1.23 | (0.66-2.27) |  | 1.16 | (0.91-1.49) |
| *, Adjusted for age, sex, health insurance type, Charlson comorbidity index, ICU use, number of hospital admission and COC index | | | | | | | | | | | |
| Grade 1 was defined patients with an FEV_1_ lower than 25% or Pa,O_2_ less than 55 mmHg; Grade 2 was defined patients with FEV_1_ lower than 30% or Pa,O_2_ less than 60mmHg ; Grade 3 was defined patients with FEV_1_ lower than 40% or Pa,O_2_ less than 65 mmHg; None was defined FEV_1_ normal and Pa,O_2_ normal . | | | | | | | | | | | |
